# A cytosolic class II small heat shock protein, PfHSP17.2, confers resistance to heat, cold, and salt stresses in transgenic *Arabidopsis*

**DOI:** 10.1590/1678-4685-GMB-2017-0206

**Published:** 2018

**Authors:** Lu Zhang, Weijuan Hu, Yike Gao, Huitang Pan, Qixiang Zhang

**Affiliations:** ^1^ Department of Landscape Architecture, School of Civil Engineering and Architecture, Zhejiang Sci-Tech University, Hangzhou, Zhejiang Province, China; ^2^ College of Landscape Architecture, Beijing Forestry University, China National Engineering Research Center for Floriculture, Beijing, China

**Keywords:** sHSPs, stress tolerance, Arabidopsis

## Abstract

We cloned and characterized the full-length coding sequence of a small heat shock (sHSP) gene, *PfHSP17.2*, from *Primula forrestii* leaves following heat stress treatment. Homology and phylogenetic analysis suggested that *PfHSP17.2* is a cytosolic class II sHSP, which was further supported by the cytosolic localization of transient expression of *PfHSP17.2* fused with green fluorescent protein reporter. Expression analysis showed that *PfHSP17.2* was highly inducible by heat stress in almost all the vegetative and generative tissues and was expressed under salt, cold, and oxidative stress conditions as well. Moreover, the expression of *PfHSP17.2* in *P. forrestii* was detected in certain developmental growth stages. Transgenic *Arabidopsis thaliana* constitutively expressing *PfHSP17.2* displayed increased thermotolerance and higher resistance to salt and cold compared with wild type plants. It is suggested that *PfHSP17.2* plays a key role in heat and other abiotic stresses.

## Introduction

Abiotic stresses such as high temperature, drought, salt, cold, and oxidative damage are the main factors impacting plant growth (Vermeulen *et al.*, 2012; Nelson *et al.*, 2014; Kumar *et al.*, 2015). In response to these stresses, plants have developed a wide range of physiological and biochemical mechanisms to protect themselves from damage (Cabane *et al.*, 1993; Sun *et al.*, 2001; Murakami *et al.*, 2004; Hu *et al.*, 2009; Ahuja *et al.*, 2010, Perez-Clemente *et al.*, 2013; Ruibal *et al.*, 2013). Heat shock proteins (HSPs), which could be induced in almost all organisms by high temperature and other abiotic and biotic stresses, were accumulated in plants as one of the strategies to deal with stressful conditions (Derocher *et al.*, 1991; Lee *et al.*, 1997; Malik *et al.*, 1999; Zhou *et al.*, 2012; Park *et al.*, 2015; Zhai *et al.*, 2016). HSPs are evolutionarily conserved proteins. They are divided into five subfamilies based on their apparent molecular weights, viz. HSP100, HSP90, HSP70, HSP60, and small HSPs (Boston *et al.*, 1996). Among them, small heat shock proteins (sHSPs), which could either protect the plant from damage caused by the stress or help repair the damage, were proved to have vital roles in response to abiotic stresses (Chao *et al.*, 2009; Lin *et al.*, 2010; Wang *et al.*, 2015). According to sequence homology, cellular compartmentation, and immunological properties, sHSPs are classified into 11 different classes: classes I–VI, localized in nucleus or cytoplasm, and the other five positioned in mitochondria, chloroplast, peroxisomes, and endoplasmic reticulum (Waters *et al.*, 2008).

Although sHSPs are highly conserved proteins of around 15 to 42 KDa and characterized by possession of a a-crystalline domain at their C-terminus, their unusual abundance and diversity in plants is considerable and the mechanism of sHSP action is not fully understood. Apart from elevated temperatures, sHSP accumulation could also be triggered by other stress conditions, such as drought, salinity, and cold, and by developmental regulation, such as seed germination, embryogenesis, and fruit development (Coca *et al.*, 1994; zur Nieden *et al.*, 1995; Sun *et al.*, 2001, 2002; Chauhan *et al.*, 2012; Koo *et al.*, 2015). The cytosolic class I and II proteins were found in the absence of stress in maturing seeds of several species such as *Nelumbo* and wheat (Efeoglu, 2009; Zhou *et al.*, 2012). It has been observed that in maize and other plants, different HSPs express abundantly during pollen development (Magnard *et al.*, 1996; Young *et al.*, 2001; Kumar *et al.*, 2015). Furthermore, overexpression of sHSPs in plants enhance their tolerance to abiotic stresses (Ahn and Zimmerman, 2006; Maimbo *et al.*, 2007; Jiang *et al.*, 2009; Chauhan *et al.*, 2012), whereas plants with reduced expression of sHSP exhibit compromised tolerance to heat resistance (Charng *et al.*, 2006) and disease (Maimbo *et al.*, 2007; Ju *et al.*, 2017).

In our previous study (Hu *et al.*, 2010), *Primula forrestii* exhibited superior thermal tolerance under heat stress compared with six other *Primula* species including *P. malacoides*, *P. obconica*, *P. veris*, *P. saxatilis*, and *P. denticulata*. Using the suppression subtractive hybridization (SSH) method, we found a sHSP gene that was remarkably up-regulated in the leaves of *P. forrestii* under high temperature treatment (42 °C for 2 h). In the present study, the full-length cDNA of this gene, termed *PfHSP17.2*, was isolated and characterized from the heat stressed leaves of *P. forrestii*. It was significantly induced by other abiotic stresses, such as salt, cold, and oxidative stress, and its expression could also be detected at certain stages of seed development and germination. Transgenic *Arabidopsis* plants overexpressing *PfHSP17.2* displayed an improved salt and temperature tolerance compared with wild type (WT) plants.

## Material and Methods

### Plant material and growth conditions

Seeds of *P. forrestii* were harvested from Xiaotangshan experimental fields in Beijing, and the original seeds were collected from Lijiang in the Yuman province in 2007. Seeds were sown in a sterilized soil incubator for germination in a greenhouse maintained at 25 ± 3 °C and at 75-80% relative humidity. Four weeks later, seedlings were transferred into black plastic pots containing 3/4 peat moss and 1/4 perlite, and watered with standard Hoagland solution every 15 d.

*Arabidopsis thaliana* (ecotype Columbia-0) plants were grown in-soil in the greenhouse (16 h light/8 h dark, 70% relative humidity, 22-25 °C) or in Petri dishes containing Murashige and Skoog (MS) medium with 4.4 gL^-1^ MS salts, 1% sucrose, and 0.8% agar, pH 5.7. In all experiments, seeds of WT and transgenic lines produced in the same culture cycle were used for analyses.

### Full-length *PfHSP17.2* cDNA cloning and bioinformatics analysis

Total RNA was isolated from *P. forrestii* treated with heat stress for 2 h using TRIZOL reagent (Invitrogen) according to the manufacturer’s instructions. cDNA library construction was performed using a SMART cDNA library construction kit (Clontech). The full-length cDNA sequence of the *PfHSP17.2* gene was directly cloned by screening the cDNA library using expressed sequence tag (EST) analysis. The functional annotation of ESTs was performed using BLASTX and BLASTN. Protein sequences from species other than *P. forrestii* were retrieved from GenBank. Then, protein sequences of PfHSP17.2 and other cytosolic class II sHSPs from various plant species were aligned by CLUSTAL W. The phylogenetic tree was constructed by MEGA3 with the neighbor-joining algorithm using default settings.

### *PfHSP17.2* gene expression profiles in response to abiotic stress

In response to different abiotic stress conditions, the expression profiles of the *PfHSP17.2* gene were analyzed using semi-quantitative RT-PCR. Total RNA was extracted from each sample using Plant RNA Extraction kit (TianGen). One μg of RNA was reverse-transcribed in a total volume of 10 μL with 1 μL oligo(dT)_18_ primer (100 pM), 1.0 μL dNTPs (10 mM), 2 μL 5 M-MLV buffer, 0.25 RNase inhibitor (40 U/μL), and 0.5 μL reverse-transcriptase M-MLV (200 U/μL). The following gene-specific primers were used: Pf2-F2 (5’-GAGAACACGGGGGAC TCTTGACCAT-3’), Pf2-R2 (5’-ATCGGGGAAATTCG AGCTGGTCACC-3’), Actin-F (5’-TCTGGCATCATAC CTTCTACA-3’), and Actin-R (5’-GGATGGCTGGAAG AGGAC-3’). The semi-quantitative RT-PCR was performed in a total volume of 25 μL with the following amplification conditions: 4 min at 94 °C; 30 cycles of 30 s at 94 °C; 40 s at 55 °C; 1 min at 72 °C; and lastly 6 min at 72 °C. PCR products were analyzed by 0.8% agarose gel electrophoresis. All the experiments were performed in triplicate.

### Subcellular location analysis

The full-length *PfHSP17.2* coding region (without the stop codon) was ligated to the 3’ end of a green fluorescent protein (GFP) to generate a PfHSP17.2–GFP fusion construct, which was driven by the cauliflower mosaic virus (CaMV) 35S promoter. An empty pCAMBIA 1302 was used as a control. The PfHSP17.2–GFP construct and 35S-GFP (control) were introduced into onion epidermal cells by particle bombardment (Bio-Rad, CA, USA). Following bombardment, the cells were incubated in the dark at 25°C for 2–3 days prior to observation. Transiently transformed cells were analyzed using a confocal laser scanning microscope.

### Stress treatments of *P. forrestii*

Two-month-old plants of *P. forrestii* were treated with half-strength MS salt solution containing either 200 mm NaCl (salt stress treatment), 20% PEG (drought stress treatment), 0.05% H_2_O_2_ (oxidative stress treatment) or 100 μM ABA. The roots, petioles, leaves, and flowers of the treated plants were collected after a 2-h treatment. For heat and cold stress treatment, two-month-old plants of *P. forrestii* grown in normal conditions were transferred to 42 °C or 4 °C for 12 h. Then, leaves were collected at 0, 10, and 20 min, and 0.5, 1, 1.5, 2, 4, 6, and 12 h after initiation of stress treatment. Plant material was placed directly into liquid nitrogen and stored at -80 °C until use.

### Generation of transgenic *Arabidopsis* plants

The sequencing-confirmed coding region of *PfHSP17.2* cDNA was cloned into the pCAMBIA1301 vector under the control of a CaMV 35S RNA promoter. The pCAMBIA1301-35S-*PfHSP17.2* recombinant vector was subsequently transferred into *Agrobacterium* GV3101 and were transformed into *Arabidopsis* wild type carried out by the floral dip method (Clough and Bent, 1998).

The seeds of T_0_ generation were harvested and sown in MS. Two-week-old seedlings of T_1_ plants were screened by hygromycin. Positive plants were transferred into soil and grown in a greenhouse at 25/22 °C (day/night) with a 16-h light/8-h dark photoperiod and 70% relative humidity. The surviving transformants (T_1_) were confirmed by PCR using the primer pairs Pf2-F1 and Pf2-R1 mentioned above. *Actin* (At)-F (5’-AGGTAATCAGTAAGGTCACGG-3’) and *Actin* (At)-R (5’-GGATGGCTGGAAGAGGAC-3’) were used to amplify the *Arabidopsis Actin* gene fragment as an internal control. T_2_ seeds were placed on MS agar medium containing hygromycin and the transgenic lines with a 3:1 segregation ratio (resistant:sensitive) were selected to produce T_3_ seeds. The T_3_ lines displaying 100% hygromycin resistance were considered homozygous and used for further experiments. The *Arabidopsis* WT was used as control. All seeds of WT and transgenic plants were collected at the same stage.

### Stress treatment of transgenic *Arabidopsis* plants

For heat stress treatment, wild type and transgenic seedlings were germinated on the same MS medium containing agar in Petri dishes. The Petri dishes were immersed in a water bath at different temperatures for heat tolerance assays. Two 10-day-old transgenic lines and a wild-type control line were exposed to 43 °C for 1 h for the basal thermotolerance assay and to 37 °C for 1 h, 22 °C for 3 h, and 47 °C for 1 h for the acquired thermotolerance assay (modified from Zhu *et al.*, 2009). About 35 plants of each genotype were used. After incubation at 22 °C for 3 d, photographs were taken and the survival rates were calculated.

For the hypocotyl thermotolerance experiment, seedlings were grown on the same MS medium plates. The plates were kept in the dark for 3 d and then transferred to 45 °C for 2 h. Subsequently, the seedlings were incubated under 22 °C. After 3 d recovery, the extent of hypocotyl elongation was measured (Hong and Vierling, 2000).

To evaluate whole plant tolerance to heat stress, 3-week-old wild-type and transgenic seedlings incubated in pots (1 plant per pot) were transferred to 42 ± 1 °C, at relative humidity of 70% for 6 h, and then returned to 22 °C for 5 d. Physiological parameters were assessed immediately after this stress treatment, while photographs were taken after a 5-d recovery (Zhang *et al.*, 2013).

For salt stress treatment, seeds from transgenic and control lines were germinated on MS plates supplemented with 0 mm NaCl, 150 mm NaCl, and 200 mm NaCl respectively. The seeds were stratified by incubation in the dark at 4 °C for 3 d prior to placement under 16-h light/8-h dark conditions with 70% relative humidity and 22-25°C. Germination rates were scored after 10 d. Seedlings were grown in MS medium for 7 d and then transferred to MS medium containing NaCl for 10 d, and the number of lateral roots and the root lengths were measured and calculated (modified from Liu *et al.*, 2011).

In addition, 7-day-old seedlings of wild type and transgenic *Arabidopsis* from MS agar medium were grown in potted soil for a week. Then, the plants were irrigated with 30 mL of a 300 mm NaCl solution for 7 d and then irrigated again for 3 d with pure water for recovery (Zhang *et al.*, 2008). Physiological parameters were assessed immediately after this stress treatment, while photographs were taken after a 7-d recovery.

For freezing stress treatment, 3-week-old transgenic and wild type *Arabidopsis* plants were exposed to -10 °C for 2 h, and then returned to normal conditions. Frequency of survival was determined and photographs were taken after 7 d. In addition, 6-week-old transgenic and control line seedlings in each of three independent experiments were incubated at 4 °C for 24 h, removed at desired freezing temperature for 30 h, thawed at 4 °C for 12 h in the dark, and then returned to the original growth condition. Physiological parameters were assessed immediately after this stress treatment (modified from Kong *et al.*, 2011).

### Measurement of electrolyte leakage, malondialdehyde (MDA), chlorophyll content, proline content, and peroxidase (POD) activity

The relative electrical conductivity (EC) was measured as described previously (Sharom *et al.*, 1994; Tang, 1999). Samples were separated into two equal groups. The first group was shaken in 5 cm^3^ double-distilled water at 170 rpm for 2 h at 25 °C, the second group was boiled for 30 min. The relative conductivity (%) was calculated as the ratio of EC of intact leaves and EC of boiled leaves 100.

The extent of lipid peroxidation was estimated by measuring the amount of MDA according to Quan *et al.* (2004). A total of 0.5 g of fresh material was ground in 5 mL of 5% (w/v) trichloroacetic acid (TCA), and 2 mL of supernatant was mixed with 2 mL of 0.67% (w/v) thiobarbituric acid (TBA) in 5% (w/v) TCA and incubated at 100°C for 30 min. After centrifuging, the optical density was measured at 450, 532, and 600 nm, respectively. The amount of MDA was calculated from the following formula: C = 6.45(A_532_–A_600_) -0.56A_450_.

After freezing treatment, chlorophyll was extracted from individual leaves with 95% ethanol, and chlorophyll contents were determined spectrophotometrically according to Lichtenthaler and Hartmut (1987). Absorbance was recorded at 645, 663, and 470 nm, and chlorophyll and carotenoid content were calculated according to the following formula:

$$\begin{array}{l} Chla=\frac{\left[\left(12.3A663-0.86A645\right)\times V\right]}{X\times 1000\times W}\\ Chlb=\frac{\left[\left(19.3A645-3.6A663\right)\times V\right]}{X\times 1000\times W}\\ Carotenoids=\frac{\left[1000\times A470-\left(1.82Cha-85.02Chb\right)\right]}{198} \end{array}$$

V = volume in mL; X = path length, 1 cm; W = fresh weight in grams.

Proline content was measured following the methods of Bates *et al.* (1973). Rosette leaves (0.5 g) were excised into 5 mL, 3% aqueous sulfosalicylic acid and incubated at 100 °C for 10 min. The homogenate was centrifuged at 12000 x *g* for 10 min. The reaction mixture containing 2 mL of supernatant, 2 mL glacial acetic acid, and 2 mL ninhydrin reagent (2.5% ninhydrin in 60% phosphoric acid) was incubated at 100 °C for 30 min and terminated by cooling the tubes on ice. The absorbance was determined at 520 nm.

To measure POD activity, leaf segments (0.5 g) were extracted on ice in 5 mL of 50 mm potassium phosphate buffer, pH 7.0, containing 1 mm ethylenediaminetetraacetic acid (EDTA) and 1% polyvinylpyrrolidone. The homogenate was centrifuged at 15,000 x *g* for 20 min at a temperature of 4 °C, and the supernatant was immediately used for the following antioxidant enzyme assays. Total POD activity was measured by monitoring the oxidation of 3,3’-dimethoxybenzidine at 470 nm (Zhang *et al.*, 2006).

The experiments were repeated independently at least three times and the results were consistent.

### Statistical analysis

Data were tested by analysis of variance using SPSS version 17.0. Significant differences were determined based on *p*<0.05 or *p*<0.01.

## Results

### PfHSP17.2 is a plant cytosolic class II sHSP

To investigate the molecular events of the *P. forrestii* heat response, a cDNA library was constructed using mRNAs isolated from 2-h heat-treated *Primula* plants. The full-length *PfHSP17.2* cDNA containing 458 bp exhibited high sequence similarities to other cytosolic class II sHSP genes from various species, according to BLAST (http://blast.ncbi.nlm.nih.gov/Blast.cgi), was predicted to encode a protein of 152 amino acids with a molecular mass of 17.2 kDa, and was designated as *PfHSP17.2* (GenBank accession number JX025006).

**Figure 1:**
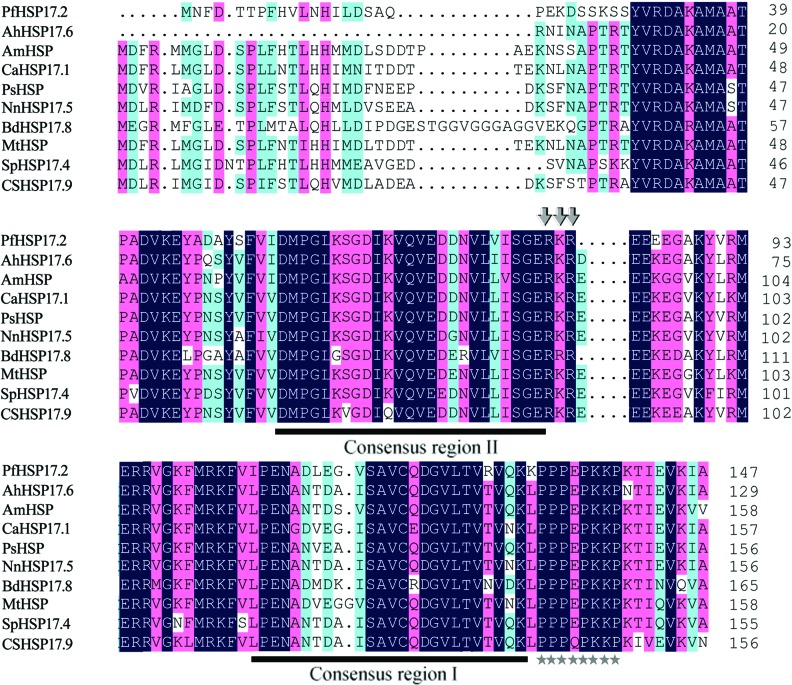
Protein sequence multiple alignment of the deduced amino acid sequence of PfHSP17.2 with other plant sHSPs. The two consensus regions in sHSPs are underlined, and identical amino acid residues are highlighted. A putative nuclear localization signal is indicated by arrows and a polyproline motif at the carboxyl end of proteins is marked with asterisks. Their GenBank accession numbers are as follows: *Primula forrestii* (PfHSP17.2; JX025006); *Arachis hypogaea* (AhHSP17.6; ACF74271.1); *Ammopiptanthus mongolicus* (AmHSP; AGS48404); *Cicer arietinum* (CaHSP17.1; XP_004501443.1); *Prunus salicina* (PsHSP; ACV93250.1); *Nelumbo nucifera* (NnHSP17.5; ABK92179.1); *Brachypodium distachyon* (BdHSP17.8; XP_003564488.1); *Medicago truncatula* (MtHSP; AES73434.1); *Solanum peruvianum* (SpHSP17.4; AAT36481.1); *Cucumis sativus* (CsHSP17.9; XP_004141334.1).

**Figure 2:**
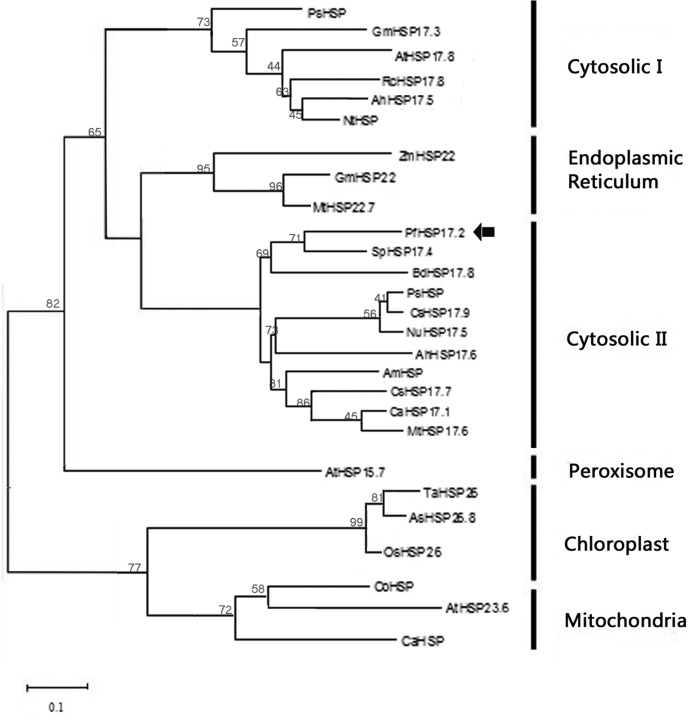
Phylogenetic analysis of the deduced amino acid sequence of *PfHSP17.2* and other small sHSPs. The gene abbreviation and GenBank accession numbers are as follows: *Primula forrestii* (PfHSP17.2; JX025006), *Arachis hypogaea* (AhHSP17.5; ABC46712.1), *Glycine max* (GmHSP17.3; NP_001235293.1), *Nicotiana tabacum* (NtHSP; ADK36668.1), *Zea mays* (ZmHSP22; NP_001151139.1), *Rosa chinensis* (RcHSP17.8; ABK32539.1), *Arabidopsis thaliana* (AtHSP17.8; NP_172220.1), *Glycine max* (GmHSP22; HSP41_SOYBN), *Medicago truncatula* (MtHSP22.7; XP_003622351.1), *Arabidopsis thaliana* (AtHSP15.7; NP_198583.1), *Triticum aestivum* (TaHSP26; AAC96315.1), *Oryza sativa* (OsHSP26; BAA78385.1), *Agrostis stolonifera* (AsHSP26.8; AAN74536.1), *Capsicum annuum* (CaHSP; ADJ57588.1), *Copaifera officinalis* (CoHSP; AEX97054.1), *Arabidopsis thaliana* (AtHSP23.6; NP_194250.1), *Arabidopsis thaliana* (AtHSP23.5; NP_199957.1); *Arachis hypogaea*(AhHSP17.6; ACF74271.1); *Ammopiptanthus mongolicus* (AmHSP; AGS48404); *Cicer arietinum* (CaHSP17.1; XP_004501443.1); *Prunus salicina* (PsHSP; ACV93250.1); *Nelumbo nucifera* (NnHSP17.5; ABK92179.1); *Brachypodium distachyon* (BdHSP17.8; XP_003564488.1); *Medicago truncatula* (MtHSP17.6; XP_003603183.1); *Solanum peruvianum* (SpHSP17.4; AAT36481.1); *Cucumis sativus* (CsHSP17.9; XP_004141334.1), *Prunus salicina* (PsHSP; ACV93250.1).

An alignment of the deduced PfHSP17.2 with other plant cytosolic class II sHSPs is shown in Figure 1. PfHSP17.2 shared high sequence identities with AhHSP17.6 (85%), AmHSP (73%), and NnHSP17.5 (71%). PfHSP17.2 contains two conserved heat shock domains, consensus I and II (Narberhaus, 2002). For plant cytosolic class II sHSPs, although a unique domain RDAKAMAATPADV is found at the N-terminal, the sequence of the N-terminal domain is divergent, which may partially account for their functional multiplicity among different plant species. Moreover, a putative nuclear localization signal (RKR) and a polyproline motif (PPPEPKKP) are found at the C-terminal of plant cytosolic class II sHSPs. A phylogenetic tree was constructed based on the similarities of 27 plant sHSPs, which separated the sHSP gene family into six clades, and *PfHSP17.2* fell into the cytosolic class II cluster (Figure 2).

### Subcellular localization assay

**Figure 3:**
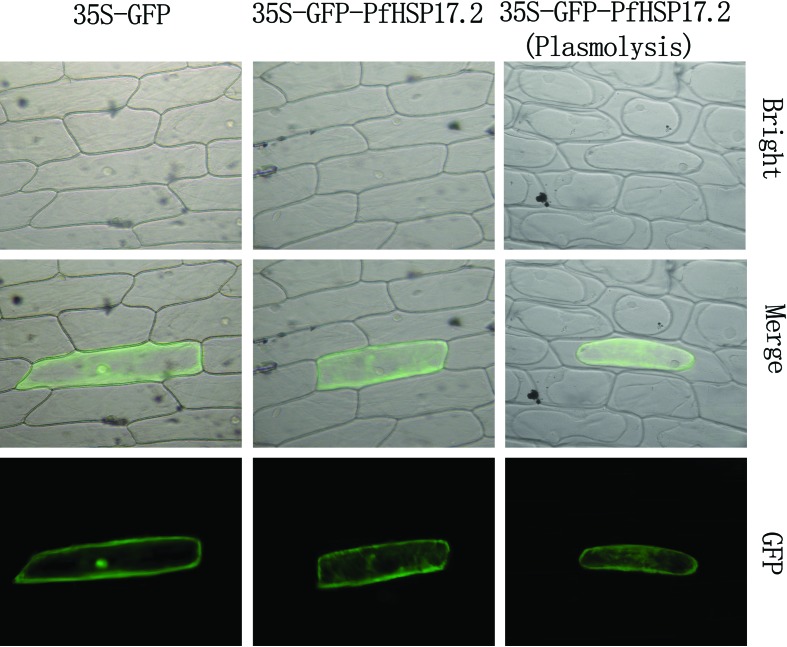
Subcellular localization of the PfHSP17.2:GFP fusion protein in onion epidermal cells. Cells were bombarded with DNA-coated gold particles carrying GFP or PfHSP17.2: GFP.

In order to examine the location of *PfHSP17.2* in the cell, a *GFP-PfHSP17.2* fusion gene construct was transformed into onion epidermal cells. As shown in Figure 3, cultured onion epidermal cells with the GFP-PfHSP17.2 fusion protein had strong fluorescence signals in the cytoplasm. In contrast, cells with the GFP fusion protein alone had signals detected in both the cytoplasm and nucleus. Therefore, *PfHSP17.2* was mainly localized in the cytoplasm.

### Expression of *PfHSP17.2* under different stress treatments

**Figure 4:**
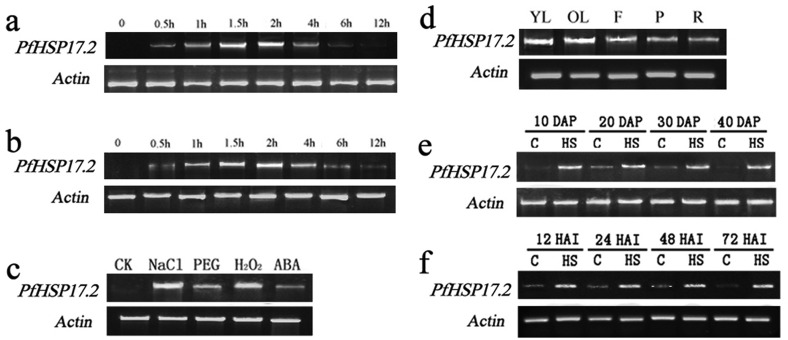
Semi-quantitative RT-PCR of *PfHSP17.2* expression in *P. forrestii.* (a) Heat treatment, time course induction of *PfHSP17.2* by heat in *P. forrestii*; (b) cold treatment, time course induction of *PfHSP17.2* by cold in *P. forrestii*; (c) Transcript levels of *PfHSP17.2* in leaves under different treatments for 3 h. CK, no treatment as control (plants only irrigated with half-strength MS salt solution); (d) expression of *PfHSP17.2* in different organs after incubation at 42 °C for 2 h; (e) semi-quantitative RT-PCR analysis in developing seeds and germinating seeds (f), respectively. DAP, days after pollination. HAI, hours after imbibition. YL, young leaves (5 d from emergence). OL, old leaves (two weeks from emergence). F, flower. P, petiole. R, root.

Upon monitoring the expression of wheat *PfHSP17.2* by semiquantitative RT-PCR at different time intervals during high temperature stress (42 °C), a transcript abundance was observed after 0.5 h of treatment, and the expression level in the leaves reached its peak at 2 h, began to decline after 4 h, and totally disappeared 12 h later (Figure 4a). Similarly, under a 4 °C cold stress, *PfHSP17.2* was expressed after 0.5 h of treatment, reaching the highest expression level 2 h later, and could still be detected after 12 h of treatment (Figure 4b). In addition, the expression of *PfHSP17.2* was examined when plants of the same age were subjected to different abiotic stress conditions, including salt stress (NaCl), osmotic stress (PEG), oxidative stress (H_2_O_2_) and ABA treatment. Increased *PfHSP17.2* transcript abundance was observed in all plant leaves analyzed, but it was slower and less abundant under drought and ABA stress treatment than that under other stressful conditions (Figure 4c). To study the tissue-specific expression pattern of *PfHSP17.2* in *P. forrestii*, its mRNAs were isolated from different tissues of the same heat-treated plant (42 °C for 2 h). The accumulation of *PfHSP17.2* was higher in the leaves relative to other tissues (petioles, roots, and flowers) (Figure 4d). Expression of the *PfHSP17.2* gene was also monitored in different stages of developing and germinating seeds separately under both thermal stressed and non-stressed conditions (Figure 4e,f). In the developing seeds, the expression of *PfHSP17.2* was high at 20 DAP and then decreased to very low levels after 40 DAP. In the germinating seeds, the expression of *PfHSP17.2* gradually decreased with the germination process from the initiation of 12 h, and it was hard to detect after 72 h. Taken together, these data indicate that *PfHSP17.2* could be specifically expressed in seeds under normal growth conditions and is strongly up-regulated in both developing and germinating seeds under heat stress.

### Overexpression of *PfHSP17.2* in transgenic plants enhances thermotolerance and resistance to salt

**Figure 5:**
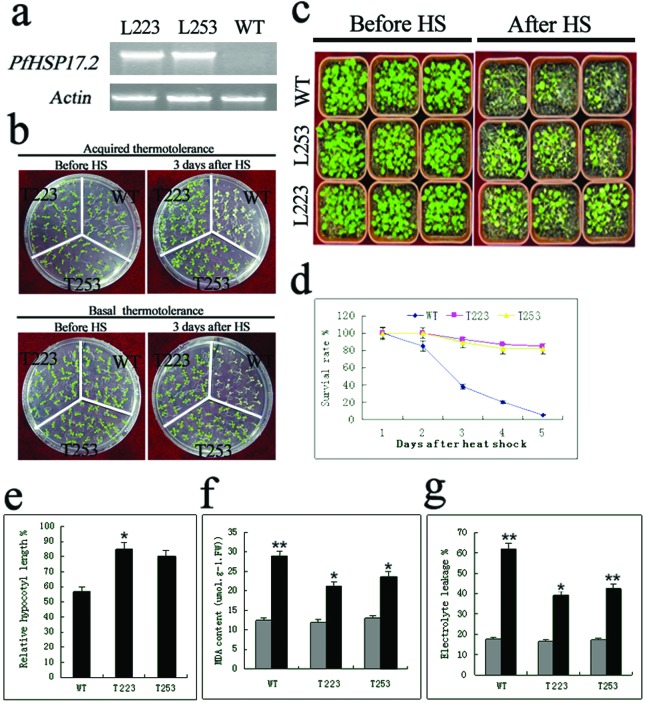
PCR analysis of *PfHSP17.2* transgenic plants and stress tolerance tests. (a) Semi-quantitative RT-PCR analysis of *PfHSP17.2* expression in WT and *PfHSP17.2* transgenic plants grown at 22°C for 4 weeks; (b) Heat treatments: aquired heat tolerance and basal heat tolerance; (c) 3-week-old plants heat treatment comparison; (d) survival rate after acquired heat treatment; (e) effect of heat stress on hypocotyl elongation of WT and *PfHSP17.2*-overexpressing plants; (f) MDA; (g) electrolyte leakage. For survival rate (d), hypocotyl elongation (e), MDA (f) and electrolyte leakage (g), error bars indicate SD (n=3). *p<0.05, ** *p*<0.01. WT, wild type; L223, L253, two transgenic *Arabidopsis* lines.

To evaluate the *in vivo* function of *PfHSP17.2*, we generated two independent T_3_ transgenic *Arabidopsis* lines, T223 and T253, which ectopically expressed *PfHSP17.2* under the control of the cauliflower mosaic virus 35S promoter. RT-PCR analysis of the 4-week-old transformants confirmed that *PfHSP17.2* was ectopically expressed in the two transgenic lines (Figure 5a).

To evaluate the effect of *PfHSP17.2* on plant HS responses, the acquired and basal thermotolerances were compared between transgenic lines and WT plants. For basal thermotolerance test, all of the WT seedlings were killed after being allowed to recover under standard conditions for 3 d, but nearly 80% of seedlings from both T223 and T253 lines survived (Figure 5b). After a conditioning pretreatment, 100% of transgenic seedlings displayed the acquired thermotolerance, but none of the WT seedlings did (Figure 5b). All of them stopped growing, their cotyledons lost chlorophyll and died after 5 d of recovery.

In order to explore the detailed morphological differences between WT and transgenic plants in thermotolerance, we monitored and compared the development of phenotype before and after heat treatment. Two lines of transgenic seedlings grew better than the WT plants after being subjected to heat stress (42°C), whereas all seedlings grew equally before the treatment (Figure 5c). The survival rate of T223 and T253 transgenic seedlings was obviously higher than that of the WT seedlings (Figure 5d). In addition, a quantitative assay was carried out to identify the thermotolerance of the transgenic plants, according to the measurement of hypocotyl elongation (Hong and Vierling, 2000). After 3 d of recovery at 22 °C, the extent of hypocotyl elongation was measured. As shown in Figure 5e, in the transgenic lines T223 and T253, the relative hypocotyl length was longer than that in the WT plants following heat shock.

To further examine the thermotolerance acquired by the transgenic plants, 4-week-old transgenic and WT seedlings grown in soil were heat-shocked at 42 °C for 12 h. The rosette leaves were taken for assays. The accumulation of MDA is often used as an indicator of lipid peroxidation (Smirnoff, 1995). At normal temperature, no significant difference in MDA content and relative electrical conductivity was detected among the WT and the transgenic lines. However, after heat stress, higher MDA content was detected in WT than in transgenic plants (Figure 5f). The relative electrical conductivity of transgenic plants was significantly lower than that of WT after high temperature stress (Figure 5g), which indicates less membrane damage in transgenic lines.

**Figure 6:**
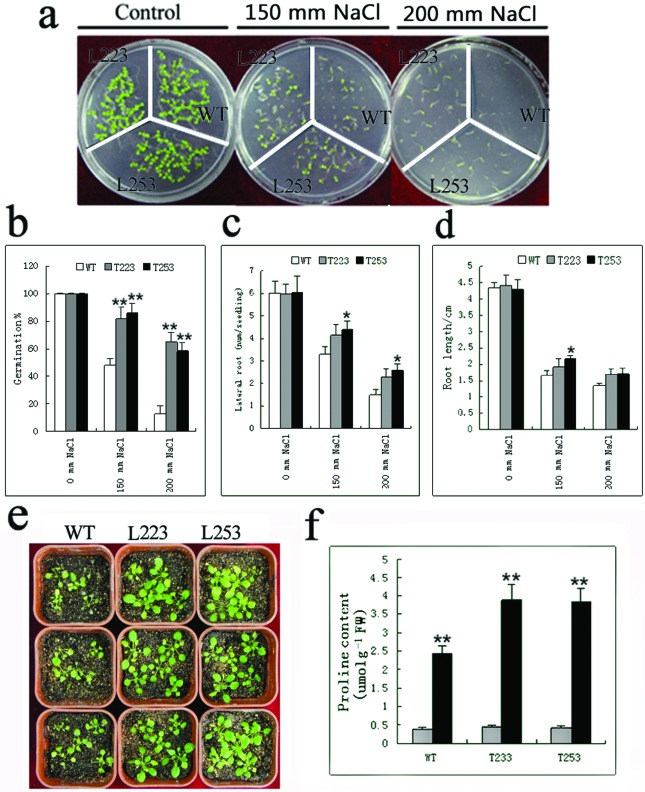
Performance of *PfHSP17.2*-overexpressing transgenic plants under salt stress. (a) Germination on different salt MS (0 mm NaCl, 150 mm NaCl, 200 mm NaCl); (b) germination rate; (c) root number; (d) root length; (e) phenotypic comparison of the plants under salt stress; (f) proline content in WT and transgenic plants in response to salt stress. Error bars indicate SD (n=3). *p<0.05, ** *p*<0.01. WT, wild type; L223, L253, two transgenic *Arabidopsis* lines.

For salt stress treatment, seeds from two homozygous *PfHSP17.2*-overexpressing lines and the WT *Arabidopsis* were germinated on MS plates supplemented with different concentrations of NaCl. On MS plates without NaCl, the transgenic lines did not show any significant difference from WT plants during germination. Compared with the wild-type *Arabidopsis*, the transgenic lines showed a significantly higher germination rate on the medium containing 150 or 200 mm NaCl (Figure 6a). More than 85% germination was found at 150 mm NaCl, compared to 50% for WT. At a high\er concentration of NaCl (200 mm) in the medium, both T223 and T253 lines still showed a germination rate of 60%, by contrast the WT seeds only germinated less than 20% (Figure 6b).

The sensitivity of seedling growth to salt stress was also examined. Seedlings grown on MS medium for 7 d were transferred to MS medium supplemented with 0, 150, and 200 mm NaCl for 10 d. Without salt treatment, all the transgenic plants showed no differences in growth compared to control plants. On medium containing 200 mm NaCl, the control seedlings showed less lateral root number than the transgenic plants (Figure 6c). However, no significant difference in root length was detected among the WT and two transgenic lines on MS medium with or without NaCl except for T253 in 150 mm NaCl (Figure 6d). In addition, 2-week-old WT and transgenic seedlings were irrigated with 300 mm NaCl solution for 7 d. The two lines of transgenic plants recovered more robustly than the WT plants after being shifted to normal conditions (Figure 6e). Proline accumulation is an important factor in determining stress tolerance and can play a major role in osmotic adjustment and may also have a number of other protective roles (Voetberg and Sharp, 1991; Liu and Zhu, 1997). At normal condition, no significant difference in proline content was detected among the WT and transgenic lines, but a significantly greater increase in the proline content was detected in the transgenic lines compared with that in the WT plants (Figure 6f), suggesting that transgenic plants were more tolerant to the salt treatment.

**Figure 7:**
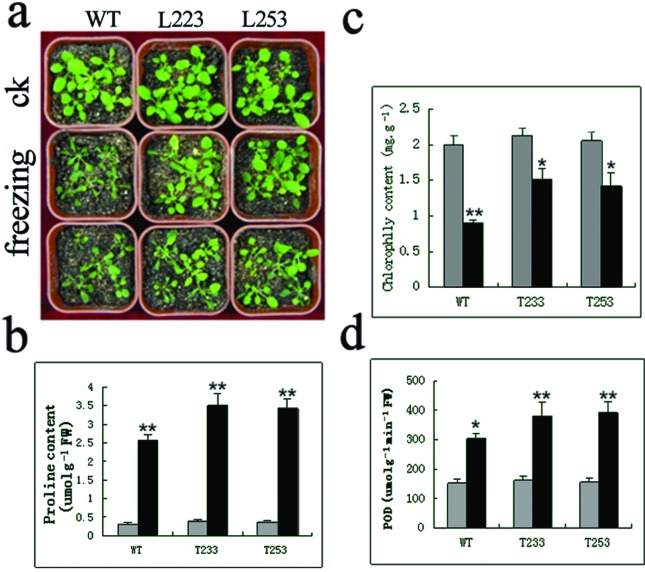
Performance of *PfHSP17.2*-overexpressing transgenic plants under cold stress. (a) Cold tolerance of the *PfHSP17.2* transformants; (b) proline content; (c) chlorophyll content; (d) POD changes. Error bars indicate SD (n=3). *p<0.05, ** *p*<0.01. WT, wild type; L223, L253, two transgenic *Arabidopsis* lines.

To test freezing tolerance of transgenic lines, after a 2 h -10°C treatment the leaves of WT plants were wilted and showed similar symptoms to that after flooding, whereas the leaves of transgenic plants turned yellow, and returned to normal growth in a short term. When returned to normal condition, transgenic plants recovered quickly, whereas the WT showed obvious tissue damage (Figure 7a). To further examine the cold tolerance acquired by the transgenic plants, 4-week-old transgenic and WT seedlings grown in soil were exposed to 4 °C for 12 h. An obviously higher proline content was detected in the transgenic lines compared with that in the WT plants (Figure 7b). Chlorophyll content assessment is one of the most useful and widely applied techniques for evaluating the effects of environmental stresses (Xue *et al.*, 2010; Kong *et al.*, 2011; Ning *et al.*, 2010). The total chlorophyll content in WT and two selected transgenic lines (T223 and T253) was remarkably decreased after stress. However, the transgenic plants’ decrease was lower than that in WT (Figure 7c), suggesting that transgenic plants are more tolerant to the chilling treatment. It has been determined previously that the activities of antioxidant enzymes in plants are correlated with increased tolerance to cold stresses (Kong *et al.*, 2011; Li *et al.*, 2012). As shown in Figure 7d, significantly higher POD activity was detected in the transgenic plants than in WT plants in response to cold conditions.

## Discussion

There have been many studies focusing on the function of sHSPs, which work as molecular chaperons and bind to unfolded proteins, prevent aggregation, induce correct refolding, and facilitate correct cell function under stress conditions (Al-Whaibi, 2011). Nevertheless, their biological functions have still not been fully understood. In the present study, we isolated and cloned a full-length cDNA of *PfHSP17.2* from heat-treated *P. forrestii* leaves. Based on alignment and phylogenetic analysis with sHSP sequences of other plants, *PfHSP17.2* was determined to be a member of the plant cytosolic class II sHSPs contained in two conserved domains, which indicated it might have similar functions as the members in cytosolic class II sHSP (Kaur *et al.*, 2015; Kumar *et al.*, 2015). Subcellular localization assay revealed that GFP-PfHSP17.2 is localized only in the cytoplasm (Figure 3), similarly to other cytosolic sHSPs (Scharf *et al.*, 2001; Zhou *et al.*, 2012).

Previous studies have described a correlation between sHSP expression and adaptation to various stress conditions in plants. A peroxisomes sHSP, *AtHSP15.7*, was strongly induced by oxidative stress as well as heat (Ma *et al.*, 2006). The rose sHSP, *RcHSP17.8*, was triggered by heat stress, drought, cold, and salt (Jiang *et al.*, 2009). *ThHSP18.3* from *Tamarix hispida* was found to be highly expressed under salt, drought, heavy metals, and both low and high temperatures (Gao *et al.*, 2012). The obvious induction of *NnHSP17.5* in *Nelumbo nucifera* resulted from heat stress and oxidative stress (Zhou *et al.*, 2012). However, an *Arabidopsis* cytosolic class II sHSP, *AtHSP17.6A*, was induced by osmotic stress but not oxidative stress (Sun *et al.*, 2001). *ZmHSP16.9C* was obviously accumulated in response to heat and oxidative stress, but could not be induced by NaCl, PEG, and cold conditions (Sun *et al.*, 2012). Similarly, the expressions of *OsHSP16.9A*, *OsHSP16.9B*, and *OsHSP16.9C* were not detected under salt, drought, and cold stresses (Guan *et al.*, 2004; Sarkar *et al.*, 2009).

In this study, the expression of *PfHSP17.2* was found rapidly induced in leaves of *P. forrstii* in response to various abiotic stresses, including heat shock (42 °C), cold (4°C), salt (NaCl), and oxidative stress (H_2_O_2_) (Figure 4a-c), indicating that *PfHSP17.2* functions as a stress response of heat as well as cold and salt. However, the expression of *PfHSP17.2* was not obviously affected by ABA treatment compared with its accumulation under other stresses mentioned above (Figure 4c). It is possible that, like *PfHSP17.1* (Zhang *et al.*, 2013), *PfHSP17.2* functions in an ABA-independent manner. Furthermore, the up-regulation of *PfHSP17.2* in the leaf of *P. forrestii* reached an expression peak within 2 h in response to both 42 °C (Figure 4a) and 4 °C temperature stresses (Figure 4b), reflecting the instantaneous and transient feature of heat shock response. However, the expression of *PfHSP17.2* could still be detected after 12 h of cold stress, while it was hard to find under 6 h of heat stress, which indicates that *PfHSP17.2* may have different mechanisms in response to different stressful conditions. In addition, different expression patterns of *PfHSP17.2* were observed in leaves, petioles, and roots (Figure 4d), which suggests that *PfHSP17.2* has tissue-specific expression patterns, and may have different functions in different tissues in response to the same stress. As shown in Figure 4d, in response to heat treatment, *PfHSP17.2* may mainly function in leaves while *PtHSP17.8* was significantly accumulated in stem and roots (Li *et al.*, 2016).

Numerous studies have previously described that sHSPs were not generally present in vegetable tissues under normal conditions, but were found at specific developmental stages, such as seed development and germination, pollen development, and fruit maturation (Waters *et al.*, 1996; He and Yang, 2013). A *Nelumbo* cytosolic class II sHSP gene (*NnHSP17.5*) was strongly expressed after 15 days of pollination. *Arabidopsis* cytosolic class I sHSPs were suggested to play a role in early stages of seed development (Dafny-Yelin *et al.*, 2008). A wheat chloroplastic small heat shock protein (sHSP26) was involved in seed maturation and germination. Similarly, the expression of *PfHSP17.2* was detected specifically in seeds under the normal physiological condition at certain stages of seed development and germination (Figure 4e,f), which is in accordance with the observations in other studies (Wehmeyer *et al.*, 1996; Sun *et al.*, 2001; Kaur *et al.*, 2015). However, it is unlike *OsHSP18.2*, which is markedly increased at the late maturation stage and is highly abundant in dry seeds, and sharply decreases after germination, indicating it may participate in seed vigor and longevity (Kaur *et al.*, 2015). Our data suggest that *PfHSP17.2* may play an important role during seed maturation and subsequent seed germination, and can thereby contribute to seed viability and vigor.

Evidence for plant sHSP stress-related functions *in vivo* has been demonstrated by the over-expression of specific sHSPs in various plants (Sanmiya *et al.*, 2004; Perez *et al.*, 2009; Xue *et al.*, 2010; Zhou *et al.*, 2012). Transgenic *Arabidopsis* that constitutively expressed RcHSP17.8 exhibited increased tolerance to heat, salt, osmotic, and drought stress (Jiang *et al.*, 2009). Overproduction of sHSP17.7 in rice enhanced drought tolerance in transgenic seedlings (Murakami *et al.*, 2004). In *Arabidopsis*, overexpression of wheat chloroplastic sHSP26 resulted in improved heat tolerance (Chauhan *et al.*, 2012). A *Gossypium arboreum* sHSP, GHSP26, enhanced drought tolerance in transformed cotton plants (Maqbool *et al.*, 2010). Overexpression of *PtHSP17.8* enhanced tolerance to heat and salt stresses in *Arabidopsis* (Li *et al.*, 2016).

In this study, the PfHSP17.2 coding sequence was introduced into *A. thaliana* by Agrobacterium-mediated transformation and overexpressed under control of the CaMV 35S promoter. Under normal growth conditions, no apparent phenotypic difference was noted in the transgenic lines T223 and T253, compared with the WT line. However, significant differences in heat stress tolerance were indicated by phenotypic changes (Figure 5b,c).

As important physiology indexes, relative electrical conductivity, proline, and chlorophyll contents, MDA and antioxidative enzymes such as POD activities could be used as indicators to evaluate stress tolerance in plants (Kar and Mishra, 1976). Transgenic plants displayed an improved thermal tolerance compared with the WT *Arabidopsis*. This response was also characterized by higher survival rate calculation, longer hypocotyl elongation measurement, less MDA, and electrolyte leakage in transgenic lines (Figure 5). These results demonstrated that transgenic *Arabidopsis* have higher heat stress tolerance than WT plants.

For salt stress, according to the measurements of the germination rate, the numbers of lateral roots, the length of roots, and proline content, two transgenic lines showed improved salt tolerance (Figure 6). In the cold stress experiment, the transgenic plants had more biomass after stress (Figure 7a), and maintained higher levels of chlorophyll content, proline content, and POD, suggesting that transgenic *Arabidopsis* plants have a higher level of tolerance to low temperature. As a molecular chaperon, PfHSP17.2 might not be involved in the biosynthesis of proline, chlorophyll content, or antioxidative enzymes directly, so they were not significantly changed in transgenic lines (T223 and T253) under normal conditions, despite the fact that *PfHSP17.2* was driven by a constitutive 35S promoter. However, the over-accumulated PfHSP17.2 could protect enzymes and proteins such as proline, to prevent their destruction or degradation under stress conditions in transgenic *Arabidopsis*, thus enhancing the stress tolerance of transgenic plants.

In summary, our results demonstrate that a plant cytosolic class II sHSP gene, *PfHSP17.2*, was remarkably induced in *P. forrstii* leaves under high temperature treatment and can also be significantly up-regulated under cold, salt, and oxidative stress conditions, as well as in the seed development and germination processes. Moreover, the overexpression of *PfHSP17.2* in *Arabidopsis* may contribute to the enhanced tolerance to thermal, salt, and cold stresses. Further investigation are needed to gain more information on the function and regulatory mechanisms of *PfHSP17.2* in plant-stress responses.
